# The significance of G-CSF expression and myeloid-derived suppressor cells in the chemoresistance of uterine cervical cancer

**DOI:** 10.1038/srep18217

**Published:** 2015-12-15

**Authors:** Mahiru Kawano, Seiji Mabuchi, Yuri Matsumoto, Tomoyuki Sasano, Ryoko Takahashi, Hiromasa Kuroda, Katsumi Kozasa, Kae Hashimoto, Aki Isobe, Kenjiro Sawada, Toshimitsu Hamasaki, Eiichi Morii, Tadashi Kimura

**Affiliations:** 1 Department of Obstetrics and Gynecology Osaka University Graduate School of Medicine; 2-2 Yamadaoka; Suita; Osaka; 565-0871; Japan; 2 Department of Biomedical Statistics Osaka University Graduate School of Medicine; 2-2 Yamadaoka; Suita; Osaka; 565-0871; Japan; 3 Department of Molecular Pathology; Osaka University Graduate School of Medicine; 2-2 Yamadaoka; Suita; Osaka; 565-0871; Japan

## Abstract

Granulocyte-colony stimulating factor (G-CSF) producing malignant tumor has been reported to occur in various organs, and has been associated with poor clinical outcome. The aim of this study is to investigate the significance of tumor G-CSF expression in the chemosensitivity of uterine cervical cancer. The clinical data of recurrent or advanced cervical cancer patients who were treated with platinum-based chemotherapy were analyzed. Clinical samples, cervical cancer cell lines, and a mouse model of cervical cancer were employed to examine the mechanisms responsible for the development of chemoresistance in G-CSF-producing cervical cancer, focusing on myeloid-derived suppressor cells (MDSC). As a result, the tumor G-CSF expression was significantly associated with increased MDSC frequencies and compromised survival. *In vitro* and *in vivo* experiments demonstrated that the increased MDSC induced by tumor-derived G-CSF is involved in the development of chemoresistance. The depletion of MDSC via splenectomy or the administration of anti-Gr-1 antibody sensitized G-CSF-producing cervical cancer to cisplatin. In conclusion, tumor G-CSF expression is an indicator of an extremely poor prognosis in cervical cancer patients that are treated with chemotherapy. Combining MDSC-targeting treatments with current standard chemotherapies might have therapeutic efficacy as a treatment for G-CSF-producing cervical cancer.

Cervical cancer, which has an annual global incidence of 530,000 new cases, is the second most common type of cancer affecting women worldwide^1^. Although most patients can be cured with treatments based on surgery and radiotherapy, a significant number eventually develop recurrent disease: the risk of recurrence is 10–20% for FIGO stages Ib-IIa and 50–70% in stages IIb-IVa^2^.

Chemotherapy is the main treatment for patients with recurrent or advanced cervical cancer, except for those who are amendable to surgical resection or salvage radiotherapy. Based on phase III trials conducted in the past few decades, platinum-based combination chemotherapies including cisplatin/paclitaxel and carboplatin/paclitaxel have become the standard regimens for recurrent or advanced cervical cancer^3^,^4^. However, patients with recurrent or advanced cervical cancer have a dismal prognosis (median survival: 10–18 months)^3^,^4^. Considering the short life expectancy of such patients, it is very important to identify factors that predict the outcome of salvage chemotherapy. Identifying patients who would not derive clinical benefit from salvage chemotherapy would at least avoid the administration of futile treatments and allow physicians to offer them the opportunity to receive other types of treatment including agents being evaluated in clinical trials or even best supportive care.

Tumor-related leukocytosis (TRL) is a paraneoplastic syndrome that is occasionally encountered in patients with malignant tumors (either at diagnosis or during the course of the disease), especially in those with advanced-stage disease^5^. According to previous reports, TRL occurs in 1–10% of patients with non-hematopoietic malignancies and is associated with a poor prognosis^5^. In uterine cervical cancer, approximately 9% and 15% of patients were incidentally found to have TRL at the time of the initial diagnosis and the diagnosis of recurrence, respectively^6^,^7^. TRL can be caused by the upregulated expression of hematological growth factors, including granulocyte-colony stimulating factor (G-CSF), granulocyte-macrophage-colony stimulating factor (GM-CSF), interleukin (IL)-1, IL-6, and tumor necrosis factor (TNF)-alpha^8^. Among these cytokines, G-CSF produced by tumor cells has recently been shown to stimulate tumor progression by facilitating tumor angiogenesis, promoting metastasis, and inducing immune suppression through the increased mobilization of myeloid-derived suppressor cells (MDSC) from the bone marrow^9^,^10^. G-CSF-producing malignant tumor has been reported to occur in various organs, and most of which has been associated with extremely poor clinical outcome^8^. We have recently reported that TRL-positive cervical cancer expresses G-CSF, is rapidly progressive, highly likely to develop resistance to radiotherapy, and is associated with recurrent or persistent disease^11^. However, the significance of tumor G-CSF expression and MDSC in the chemosensitivity of cervical cancer have never been investigated.

In the current study, we examined the prognostic significance of tumor G-CSF expression in patients with recurrent or metastatic cervical cancer that had been treated with platinum-based chemotherapy. Moreover, after investigating the underlying causative mechanism in *in vitro* and *in vivo* experimental models we proposed novel treatment strategies for overcoming the chemoresistance of G-CSF-producing cervical cancer.

## Results

### Clinical implications of tumor G-CSF expression in cervical cancer patients treated with platinum-based chemotherapy

**Figure 1:**
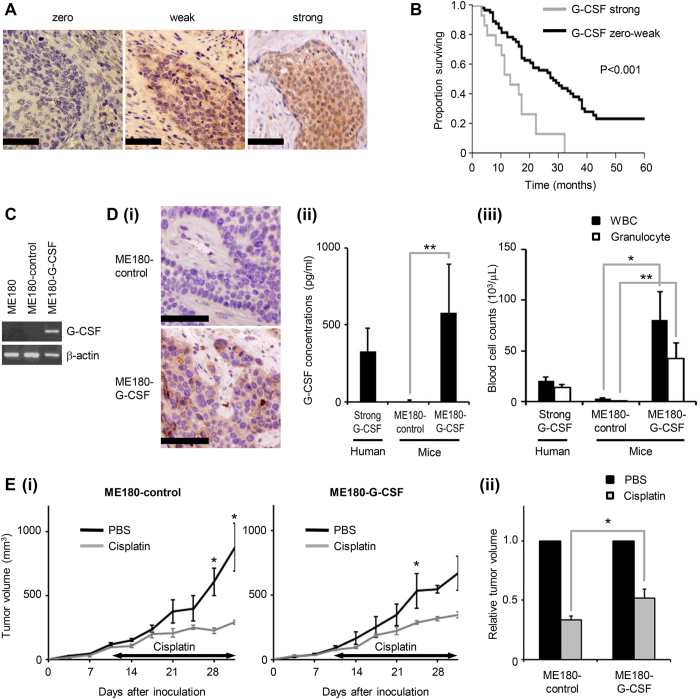
Clinical implications of tumor G-CSF expression in cervical cancer patients receiving chemotherapy. (A) G-CSF expression in cervical cancer. Cervical cancer biopsy samples were stained with anti-G-CSF antibody. Representative photographs of tumors which exhibited zero, weak and strong G-CSF expression (magnification: ×200, bar = 50 μm). (**B**) Kaplan–Meier estimates of overall survival after chemotherapy according to G-CSF immunoreactivity. Log-rank test was used to determine statistical significance. (**C**) Establishment of G-CSF-producing cervical cancer cell lines. RT-PCR analysis of the G-CSF and β-actin mRNA levels of ME180 cells that had been stably transfected with the G-CSF vector (ME180-G-CSF) or the control vector (ME180-control). (**D**) A mouse model of G-CSF-producing cervical cancer. Mice were inoculated with ME180-G-CSF (n = 5) or ME180-control cells (n = 5). Three weeks after the inoculation, their subcutaneous tumors and blood were collected for evaluation. Blood samples were also collected from G-CSF-positive cervical cancer patients (n = 6) (normal range of the serum G-CSF level in humans: <20.0  pg/mL). (i) G-CSF expression in the subcutaneous tumors. (magnification: ×200, bar = 50 μm) (ii) Serum G-CSF concentrations according to ELISA. Bars, SD. **P < 0.01, Two-sided Student’s *t* test. (iii) WBC/granulocyte counts. Bars, SD. *P < 0.05, **P < 0.01, Two-sided Student’s *t* test. (**E**) Cisplatin-resistant nature of G-CSF-producing cervical cancer. Mice that had been inoculated with the ME180-G-CSF or ME180-control cells were treated with 4 mg/kg of weekly cisplatin or PBS (n = 5 for each group). (i) Growth curves. Bars, SD. *P < 0.05 for cisplatin vs. PBS, Wilcoxon rank sum test. (ii) Relative tumor volume three weeks after chemotherapy. Bars, SD. *P < 0.05, Wilcoxon rank sum test.

A total of 82 cervical cancer patients who had been treated with platinum-based chemotherapy were included in the current study. Clinicopathological characteristics of these patients are shown in Supplementary Table S1. To investigate the G-CSF expression in cervical cancer, using the biopsy samples, immunohistochemical staining with a specific antibody against human G-CSF was performed (Fig. 1A). G-CSF expression was observed in 70 patients (85.4%). Of these, 15 patients showed strong G-CSF expression and 55 patients showed weak G-CSF expression. To understand the clinical significance of G-CSF expression in cervical cancer patients who are treated with chemotherapy, we next evaluated the associations between G-CSF immunoreactivity and the response rate or survival after chemotherapy. As shown in Fig. 1B, a significant difference in overall survival (OS) was detected between the patients that exhibited zero to weak G-CSF expression (the G-CSF-negative cervical cancer patients) and those that demonstrated strong G-CSF expression (the G-CSF-positive cervical cancer patients) (OS: 13 vs. 28 months, p = 0.0006). Of a total of 82 patients, 17 displayed complete responses (CR) and 13 exhibited partial responses (PR) to platinum-based chemotherapy. The response rate of the G-CSF-positive cervical cancer patients was 27%, which was slightly lower than that of the G-CSF-negative cervical cancer patients (39%, p = 0.3686). In a survival analysis of the responders (PR + CR), the G-CSF-positive cervical cancer patients displayed significantly shorter OS than the G-CSF-negative cervical cancer patients (OS: 24.5 months vs. 62 months, p = 0.0341) (Supplementary Figure S1A). Moreover, the G-CSF-positive cervical cancer patients exhibited marginally shorter progression-free survival (PFS) than the G-CSF-negative cervical cancer patients (median PFS: 8.5 months vs. 20 months, p = 0.0687), indicating that the responses of the G-CSF-positive cervical cancer patients did not last as long as those of G-CSF-negative cervical cancer patients (Supplementary Figure S1A). In the multivariate analysis (Table 1), strong G-CSF expression remained an independent predictor of compromised survival (P = 0.0061).

### Mouse model of G-CSF-producing cervical cancer

To establish a mouse model of G-CSF producing cervical cancer, we inoculated nude mice with cervical cancer cells that had been stably transfected with G-CSF (Fig. 1C). The expression and stimulatory activity of G-CSF in these cells were verified *in vitro* and *in vivo* (Fig. 1D). Using this mouse model, we next investigated the sensitivity of G-CSF-producing cervical cancer to cisplatin, a key anti-cancer agent in the treatment of cervical cancer. Consistent with our findings in humans, the ME180-G-CSF-derived tumors were less sensitive to cisplatin than the ME180-control-derived tumors (Figs 1E,5B,C).

### G-CSF-induced MDSC are responsible for the development of cisplatin resistance in cervical cancer

**Figure 2:**
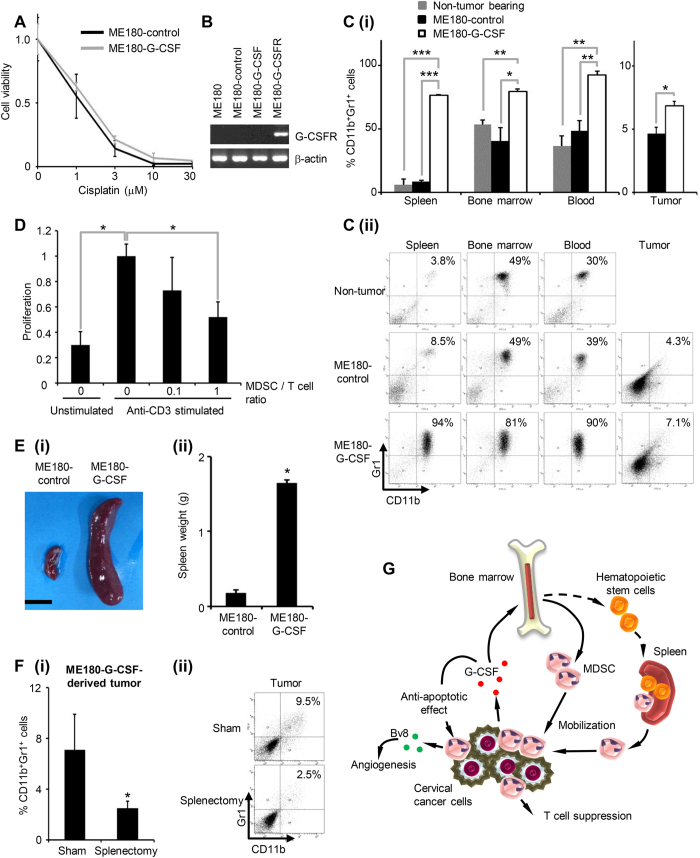
MDSC accumulation in G-CSF-producing cervical cancer derived tumor bearing mice. (**A**) *In vitro* sensitivity of cervical cancer cells to cisplatin. ME180-G-CSF or ME180-control cells were exposed to the indicated dose of cisplatin for 72 hours. Cell viability was assessed using the MTS assay. Data points, mean values; bars, SD. Data are shown as the means of triplicate samples. (**B**) RT-PCR analysis of the G-CSFR and β-actin mRNA levels of the cervical cancer cells. ME180-G-CSFR cells, into which a G-CSFR expression vector had been stably transfected, were used as positive controls. (**C**) CD11b^+^ Gr-1^+^ cell populations in G-CSF-producing cervical cancer. Mice were inoculated with ME180-G-CSF (n = 6), ME180-control cells (n = 6), or PBS alone (n = 6). Four weeks after the inoculation, their spleen, bone marrow, blood, and tumors were collected. (i) CD11b^+^ Gr-1^+^ cell populations were counted by flow cytometry. Bars, SD. *P < 0.05, **P < 0.01, ***P < 0.001, Two-sided Student’s *t* test. (ii) Representative dot plot. The percentage of the CD11b^+^ Gr-1^+^ cell is indicated. (**D**) Ability of G-CSF-induced CD11b^+^ Gr-1^+^ cells to suppress anti-CD3 mAb-stimulated T cells. Balb/c mice were subcutaneously treated with 10 μg recombinant human G-CSF for three days. CD11b^+^ Gr-1^+^ cells were isolated from their spleen. CD8^+^ T cells (2 × 10^5^ cells/well) were isolated from syngeneic mice and co-cultured with CD11b^+^ Gr-1^+^ cells at indicated ratios. Cells were incubated for 72 hours, after which BrdU was added for an additional 24 hours. T cell proliferation was determined by BrdU incorporation. Bars, SD. *P < 0.05, Two-sided Student’s *t* test. (**E**) Splenomegaly in G-CSF-producing cervical cancer. Mice were inoculated with ME180-G-CSF (n = 6) or ME180-control cells (n = 6). Their spleens were collected 4 weeks later. (i) Representative photos of the spleen (bar = 1 cm). (ii) Spleen weight. Bars, SD. *P < 0.05, Wilcoxon rank sum test. (**F**) The effect of splenectomy on MDSC accumulation in ME180-G-CSF-derived tumors. Mice that underwent splenectomy (n = 5) or sham surgery (n = 5) were inoculated with ME180-G-CSF. Three weeks after the inoculation, their subcutaneous tumors were collected. (i) CD11b^+^ Gr-1^+^ cell populations in tumors. Bars, SD. *P < 0.05, Two-sided Student *t* test. (ii) Representative data. (**G**) Proposed paracrine mechanism responsible for the chemoresistance in cervical cancer.

To investigate the mechanism of chemoresistance in G-CSF-producing cervical cancer, we first examined whether G-CSF acts via an autocrine mechanism. As shown, the ME180-control and ME180-G-CSF cells displayed similar cisplatin-sensitivity *in vitro* (Fig. 2A), which is consistent with the fact that the ME180 cells were negative for the G-CSF receptor (G-CSFR) (Fig. 2B). This result indicates that cancer cell-derived G-CSF does not have a direct stimulatory effect on cervical cancer cells. Then, we next examined whether G-CSF acts via a paracrine mechanism. As shown, the ME180-G-CSF-derived tumor-bearing mice displayed markedly increased MDSC (CD11b^+^ Gr-1^+^ cells) frequencies in their bone marrow, blood, spleens, and tumors compared with the ME180-control-derived tumor-bearing mice (Fig. 2C). The CD11b^+^ Gr-1^+^ cells isolated from the spleen of the mice significantly inhibit the T cell proliferation (Fig. 2D), which is consistent with the immunosuppressive nature of MDSC.

As the ME180-G-CSF-derived tumor-bearing mice exhibited splenomegaly (Fig. 2E), we next investigated the role of the spleen in the production of MDSC in our mouse model. The removal of the spleen significantly reduced the number of MDSC in the ME180-G-CSF-derived tumors (Fig. 2F), indicating that the spleen acts as a supplier of MDSC (Fig. 2G), which is consistent with the previous notion of a “splenic reservoir”^11^,^12^. Consistent with the findings obtained in Fig. 2C, treatment of mice with recombinant human G-CSF protein significantly increased the volume of the spleen and the frequencies of MDSC in their bone marrow, blood, and spleen (Supplementary Figure S2A).

**Figure 3:**
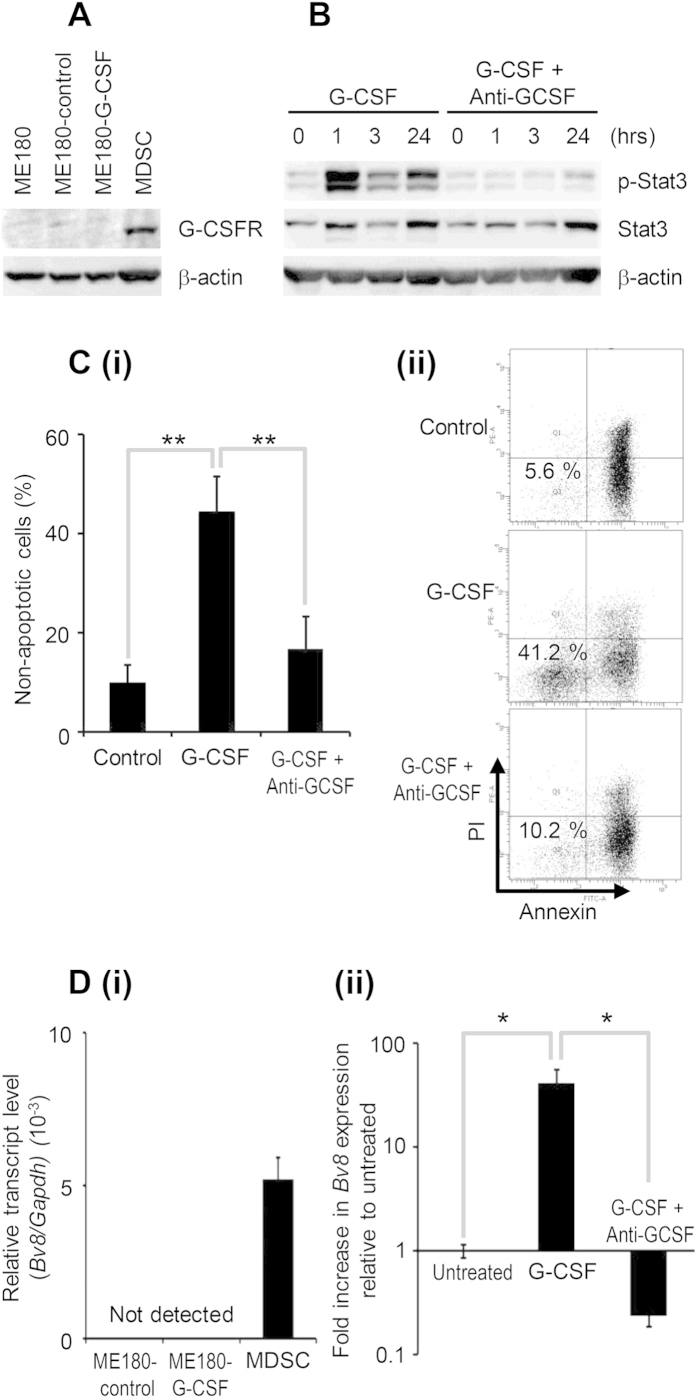
Mechanism of MDSC-mediated cisplatin resistance. (**A**) Western blot analysis of G-CSFR and β-actin expression in MDSC. (**B**) The effect of G-CSF on Stat3 activation of MDSC. MDSC were treated with 10 ng/mL G-CSF in the presence or absence of 100 μg/mL anti-G-CSF-neutralizing antibody. (i) Cells were cultured for indicated time and then activation of Stat3 in MDSC was assessed by Western blotting. (**C**) The effect of G-CSF on the survival of MDSC. MDSC were treated with 10 ng/mL G-CSF in the presence or absence of 100 μg/mL anti-G-CSF-neutralizing antibody. (i) Cells were cultured for 24 hours and then assayed for apoptosis by flow cytometry. The pooled data indicating the non-apoptotic cells were shown. Bars, SD. **P < 0.01, Two-sided Student’s *t* test. (ii) Representative dot plot. The percentage of the non-apoptotic cells is indicated. (**D**) The effect of G-CSF on the expression of Bv8 in MDSC. (i) Cervical cancer cells and MDSC were harvested, and their Bv8 expression was assessed by real-time RT-PCR. The expression level of Bv8 mRNA was normalized to that of GAPDH mRNA. (ii) MDSC were treated with 10 ng/mL G-CSF in the presence or absence of 100 μg/mL anti-G-CSF-neutralizing antibody for 4 hours. Then their Bv8 expression was assessed. Bars, 95% confidence interval. *P < 0.05, Two-sided Student’s *t* test.

As it has been reported that G-CSF inhibits the spontaneous neutrophil apoptosis through the induction of Janus kinase 2 (JAK2)- Signal transducer and activator of transcription 3 (Stat3) pathway^13^, we next investigated the effect of G-CSF on Stat3 pathway activation in MDSC and the survival of MDSC. As shown, G-CSFR expressions were observed in the MDSC (Fig. 3A), and the treatment of MDSC with human recombinant G-CSF induced the significant activation of Stat3 (Fig. 3B). Moreover, G-CSF significantly attenuated the induction of spontaneous MDSC apoptosis (Fig. 3C), indicating that G-CSF increases the number of MDSC not only by inducing the production of MDSC from bone marrow and spleen but also by prolonging their survival.

We next investigated the mechanism by which MDSC impair the efficacy of chemotherapy with focuses on the tumor-angiogenesis. As an unregulated tumor-angiogenesis is known to increase the interstitial fluid pressure and reduces the chemosensitivity of human malignancies including uterine cervical cancer^14^,^15^, we next investigated the role of the MDSC in the expression of Bv8, a potent pro-angiogenic factor that stimulates the proliferation, survival, and migration of endothelial cells^16^. As shown, Bv8 expressions were observed in the MDSC isolated from the ME180-G-CSF-derived tumor bearing mice (Fig. 3D). Moreover, the expression of Bv8 in MDSC was significantly increased in response to G-CSF treatment (Fig. 3D). As we have recently demonstrated that MDSC induced by tumor-derived G-CSF stimulate tumor-angiogenesis^11^, these results suggest that tumor-derived G-CSF and G-CSF-induced MDSC might promote cisplatin resistance, at least in part, by the stimulation of tumor-angiogenesis through the expression of Bv8.

Collectively, these results suggest that tumor-derived G-CSF is involved in the cisplatin resistance in cervical cancer by inducing the production of MDSC, prolonging their survival, and increasing their activities against the cancer cells and the cancer microenvironment (Fig. 2G).

**Figure 4:**
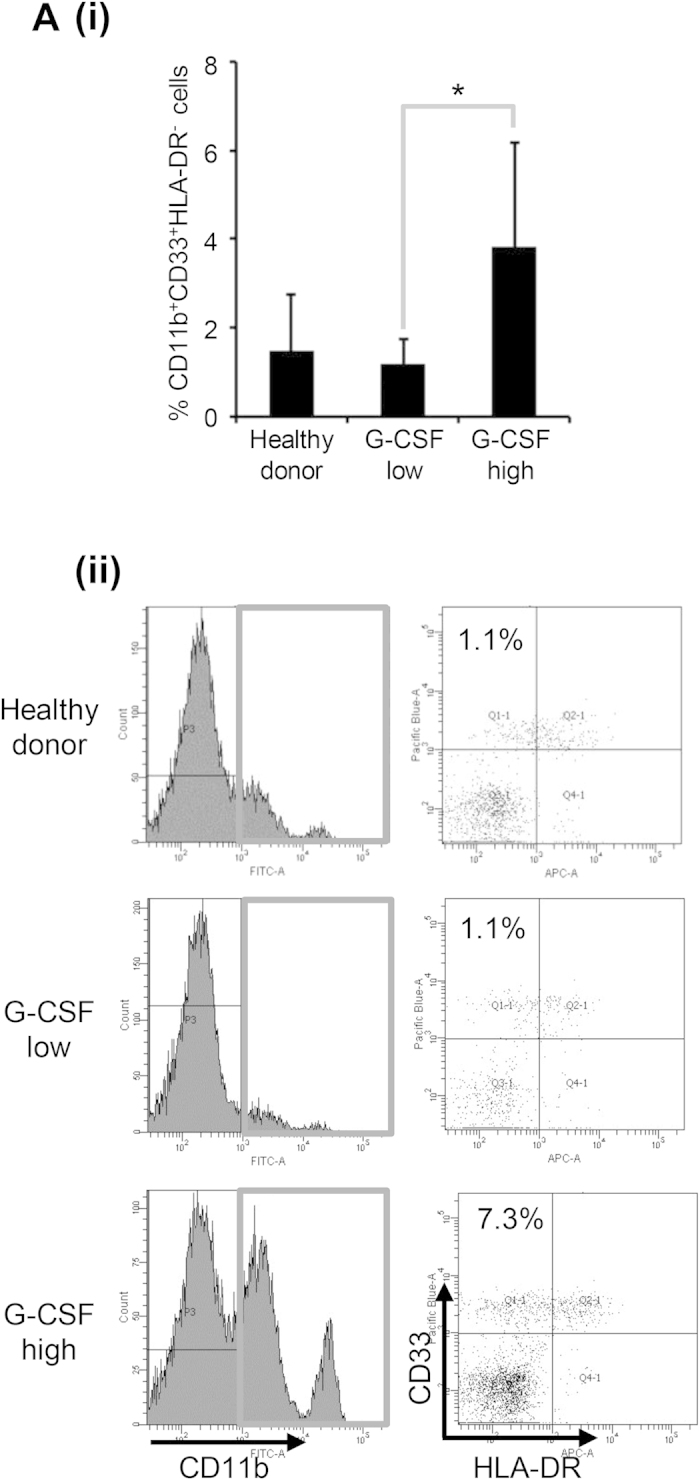
MDSC in cervical cancer patients. (**A**) Circulating MDSC levels of the cervical cancer patients. Peripheral blood mononuclear cells (PBMC) were obtained from healthy donors (n = 10), cervical cancer patients with normal G-CSF levels (n = 9) and cervical cancer patients with elevated G-CSF levels (n = 5). (i) Human MDSC, which were defined as CD11b^+^ CD33^+^ HLA-DR^−^ cells, were counted using flow cytometry. Bars, SD. *P < 0.05, Wilcoxon rank sum test. (ii) Representative dot plot. The percentage of the CD11b^+^ CD33^+^ HLA-DR^−^ cell is indicated.

To determine whether the findings we obtained in mice are representative of the clinical status of cervical cancer patients, we investigated the association between the serum G-CSF levels and the numbers of MDSC in the blood of patients with newly diagnosed cervical cancer and the healthy donors. Of the 14 women examined, 5 cervical cancer patients had elevated serum G-CSF levels (reference range, <20.0 pg/mL). Consistent with the findings obtained in mice, the peripheral blood of the cervical cancer patients with elevated serum G-CSF levels contained significantly higher numbers of MDSC; i.e., CD11b^+^ CD33^+^ HLA-DR^−^ cells. In contrast, we only detected very low numbers of MDSC in the blood of the healthy donors and the cervical cancer patients with normal serum G-CSF levels (Fig. 4A).

### MDSC depletion inhibits tumor growth and enhances the therapeutic efficacy of chemotherapy

**Figure 5:**
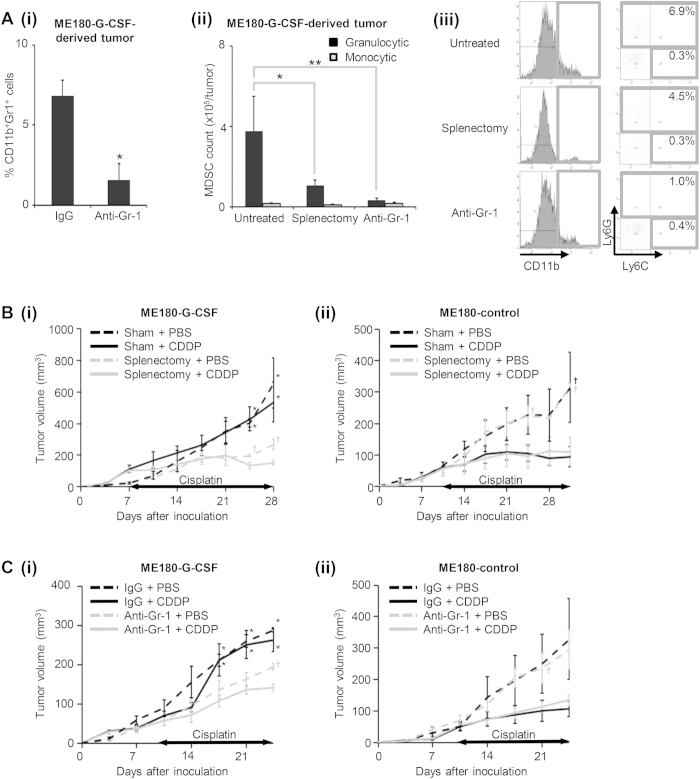
MDSC depletion and cisplatin resistance. (**A**) (i) The effects of anti-Gr-1 neutralizing antibody on MDSC accumulation in ME180-G-CSF-derived tumors. Mice that had been inoculated with ME180-G-CSF cells were treated with anti-Gr-1 neutralizing antibody (n = 6) or control IgG (n = 6) every 2 days. Three weeks after inoculation, the subcutaneous were collected for evaluation. Bars, SD. *P < 0.05, Two-sided Student *t* test. (ii–iii) The effects of splenectomy and anti-Gr-1-neutralizing antibody on MDSC subsets in ME180-G-CSF-derived tumor-bearing mice that had undergone splenectomy (n = 5), anti-Gr-1 antibody treatment (n = 5), or no treatment (n = 5). CD11b^+^ cells were gated and then re-plotted for their Ly6G and Ly6C expression to determine the frequencies of the granulocytic and monocytic MDSC subsets. (ii) Each MDSC subpopulation was counted by flow cytometry. Bars, SD. *P < 0.05, **P < 0.01, Wilcoxon rank sum test. (iii) Representative histograms and dot plots. The percentage of granulocytic and monocytic MDSC subsets is indicated. (**B**) Effects of spleen removal on the cisplatin-sensitivity of cervical cancer. Mice that had undergone splenectomy or sham surgery were inoculated with cervical cancer cells (n = 5 for each group) and treated with cisplatin or PBS. Growth curves of (i) ME180-G-CSF- and (ii) ME180-control-derived tumors. Bars, SD. *P < 0.05 for splenectomy vs sham group, ^†^P < 0.05 for cisplatin vs PBS group, Wilcoxon rank sum test. (**C**) Effects of anti-Gr-1-neutralizing antibody on the cisplatin-sensitivity of cervical cancer. Mice carrying cervical cancer-derived tumors were treated with cisplatin or PBS in combination with anti-Gr-1-neutralizing antibody or IgG (n = 5 for each group). Growth curves of (i) ME180-G-CSF- and (ii) ME180-control-derived tumors. Bars, SD. *P < 0.05 for anti-Gr-1 vs IgG group, ^†^P < 0.05 for cisplatin vs PBS group, Wilcoxon rank sum test.

To directly demonstrate the involvement of MDSC in the development of cisplatin resistance in G-CSF-producing cervical cancer, we next examined the effect of MDSC depletion on the anti-tumor effects of cisplatin *in vivo*. As shown, splenectomy (Fig. 2F) or treatment with anti-Gr-1 antibody (Fig. 5A (i)) significantly reduced the frequencies of MDSC; i.e., CD11b^+^ Gr-1^+^ cells. As CD11b^+^ Gr-1^+^ cells represent a heterogeneous population that includes granulocytic and monocytic MDSC, we further investigated the effects of splenectomy or anti-Gr-1 antibody treatment on the subsets of MDSC. As shown in Fig. 5A (ii)–(iii), we observed a marked enrichment of Ly6C^low^Ly6G^+^ granulocytic MDSC in the ME180-G-CSF-derived tumor-bearing mice, indicating that granulocytic MDSC are the dominant subset that is expanded by the G-CSF secreted by ME180-G-CSF-derived tumors. Treatment with anti-Gr-1 antibody or splenectomy significantly reduced the frequency of granulocytic MDSC, but the effects of these treatments on monocytic MDSC were minimal. As shown in Fig. 5B (i) and Supplementary Figure S3A, the ME180-G-CSF-derived tumors that developed in the splenectomized mice were significantly more sensitive to cisplatin than those that developed in the mice that underwent sham surgery. However, the effect of splenectomy was minimal in the ME180-control-derived tumor-bearing mice (Fig. 5B (ii)). Moreover, compared with the control IgG treatment, the administration of anti-Gr-1-neutralizing antibody significantly increased the sensitivity of their tumors to cisplatin (Fig. 5C (i), Supplementary Figure S3B,C). In contrast, the effect of anti-Gr-1-neutralizing antibody was minimal in the ME180-control-derived tumor-bearing mice (Fig. 5C (ii)).

## Discussion

This study showed, for the first time, that tumor G-CSF expression is associated with significantly shorter survival in cervical cancer patients receiving platinum-based chemotherapy (Fig. 1B, Table 1). Among our patients, the response rate of the G-CSF-positive cervical cancer patients was 27%, which was lower, but not significantly, than that of the G-CSF-negative cervical cancer patients (39%, p = 0.3686). However, in a survival analysis of the responders (Supplementary Figure S1A), the G-CSF-positive cervical cancer patients exhibited shorter PFS and OS than the G-CSF-negative cervical cancer patients (median PFS: 8.5 months vs. 20 months, p = 0.0687; median OS: 24.5 months vs. 62 months, p = 0.0341), indicating that the responses of the G-CSF-positive cervical cancer patients did not last as long as those of the G-CSF-negative cervical cancer patients. This is consistent with the findings of a previous report, in which we described a case of recurrent G-CSF-producing cervical cancer that progressed rapidly after initially exhibiting a significant response to platinum-based chemotherapy^17^. Importantly, a significantly positive correlation between tumor G-CSF expression and their WBC counts was observed in these patients (data not shown). Although our study was retrospective, these findings might have important clinical implications: by performing immunostaining or more simple and low-cost examinations of the peripheral blood, it might be possible to identify patients who would not derive clinical benefit from platinum-based chemotherapies and offer them the opportunity to receive other types of treatment including agents being evaluated in clinical trials or even best supportive care.

MDSC are a heterogeneous family of immature myeloid cells whose differentiation has been arrested, which can be induced by a variety of factors^18^. In previous functional studies, MDSC enhanced tumor progression in experimental mouse models by suppressing T cell function^19^, and other studies have demonstrated that an increased number of circulating MDSC is associated with advanced clinical stage, tumor burden, and survival in patients with cancers of various origins^20^,^21^. MDSC have also been shown to impair the efficacy of cancer immunotherapy^22^. However, the role of MDSC in cancer chemosensitivity has yet to be elucidated. The present study found that G-CSF-induced MDSC accumulation in tumors is responsible for the chemoresistant nature of G-CSF-producing cervical cancer: the tumor-derived G-CSF stimulates the production of MDSC, prolongs the survival of MDSC, and enhances the production of Bv8, a pro-angiogenic molecule. Thus, MDSC represent an attractive therapeutic target, and MDSC-targeting treatments might improve the prognosis of patients with G-CSF-producing cervical cancer. Previous studies have suggested that both murine and human MDSC suppress T cell functions. However, they express different surface markers, and they are induced by different mechanisms^23^,^24^. In the current study, due to the limited number of patients that agreed to undergo blood tests for MDSC, we could not draw any definitive conclusions about the association between the frequencies of each MDSC subset and the treatment outcomes of platinum-based chemotherapy. Thus, the role of human MDSC in the chemosensitivity of cervical cancer to platinum-based chemotherapy needs to be investigated further in larger, prospective clinical studies.

According to previous studies, the majority of MDSC reside in the bone marrow, and only small numbers of these cells are found in the blood and spleen in healthy individuals. MDSC markedly expand systemically in response to acute inflammation or cancer development as part of the host immune response^18^. During this process, the spleen has been shown to act as an extramedullary reservoir of MDSC, which can be mobilized to contribute to the host response^12^. Consistent with these results, we found that the removal of the spleen significantly abrogated G-CSF-induced MDSC accumulation in tumors and enhanced the efficacy of cisplatin in mice (Figs 2F and 5B, Supplementary Figure S3A), indicating that the spleen also acts as an MDSC reservoir in our mouse model, and hence, is a potential therapeutic target for treatments that aim to enhance the effectiveness of chemotherapy in G-CSF-producing cervical cancer. In a recent study of uterine cervical cancer, the TRL-positive patients had significantly larger spleens than the TRL-negative patients^11^. Splenectomy and spleen irradiation have been used as palliative treatments for patients with hematological malignancies^25^. Although it has not been confirmed whether the spleen acts as a reservoir of MDSC in humans, the results of the current study strongly indicate that the efficacy of combining spleen-targeting therapies with platinum-based chemotherapy is worth investigating in future clinical trials involving G-CSF-producing cervical cancer patients.

We also found that treatment with anti-Gr-1-neutralizing antibodies enhanced the sensitivity of G-CSF-producing cervical cancer to cisplatin (Fig. 5C, Supplementary Figure S3B,C). In preclinical studies of lung and colon carcinoma, it was reported that the therapeutic efficacy of cyclophosphamide was enhanced by MDSC depletion^26^. This indicates that the anti-tumor efficacy-enhancing effects of MDSC depletion are not specific to cisplatin. Although in humans myeloid-derived cells do not express the myeloid-cell lineage differentiation antigen Gr-1, the results of the current study indicate that pharmacological approaches that target MDSC might be effective against G-CSF-producing cervical cancer. One possible strategy of MDSC inhibition is the use of gemcitabine, which has been used in the clinical setting to treat human malignancies including uterine cervical cancer^27^. In a previous study, treatment with gemcitabine reduced the number of MDSC in mouse models of lung cancer^28^. Sunitinib, a receptor tyrosine kinase inhibitor, has also been shown to inhibit MDSC accumulation, and hence, reduce the immunosuppressive activity of MDSC, in mouse models of colon and lung cancer^29^ and human renal cell carcinoma patients^30^. Thus, we consider that the efficacy of combining MDSC-targeting therapies with conventional platinum-based chemotherapy is worth investigating in future clinical trials. The limitations of our study need to be addressed. First, we employed an anti-Gr-1 antibody and splenectomy to deplete MDSC in the current study. However, we cannot rule out the possibility that the effects of these procedures are not specific to MDSC and that they affect other cells such as neutrophils. Second, we have to recognize that it is difficult to directly demonstrate the chemoresistant nature of G-CSF-positive cervical cancer based on the data obtained in the current study, as we cannot completely eliminate the influence of the rapidly progressive nature of G-CSF-positive cervical cancer. Third, the precise mechanisms by which MDSC mediate cisplatin resistance were not fully elucidated in the current study. Fourth, although the current study focused on the “tumor-derived G-CSF-MDSC axis”, other tumor-derived factors may also play roles in the expansion of MDSC in a G-CSF-independent manner. A recent report suggested the possibility that cervical cancer cell-derived IL-6, IL-8 or CXCL1 are involved in the accumulation of CD11b^+^ Gr-1^+^ cells in tumor^31^. They also suggested that other myeloid derived cells such as macrophages in tumor stroma might be stimulated by tumor-derived cytokines^31^. Thus, we cannot exclude the possibility that other stromal cells in the tumor microenvironment might be stimulated by tumor-derived G-CSF to enhance chemoresistance of uterine cervical cancer. Accordingly, the mechanism by which MDSC is regulated and the mechanism by which G-CSF promotes chemoresistance should be investigated further. Fifth, our *in vitro* and *in vivo* studies were conducted using a single cell line (ME180) and related transfectants. As Stone *et al.* reported HPV positive cervical cancer cell lines expressed significantly higher levels of cytokines than HPV negative cells^31^. This indicates that the pattern of cytokine expression may differ depending on cervical cancer cell lines. Thus, further investigations using various cervical cancer cell lines will be required to validate our preclinical findings. Lastly, due to the retrospective nature of our clinical study, the relatively small number of patients enrolled, and the fact that it was conducted at a single institution, we cannot draw definitive conclusions regarding the predictive value of pretreatment leukocytosis in cervical cancer patients receiving chemotherapy. To validate our clinical findings, a collaborative multi-institutional investigation needs to be conducted, preferably in a prospective setting.

G-CSF is widely used during chemotherapy to reduce the risk of chemotherapy-induced neutropenia and facilitate the delivery of maximally effective doses of cytotoxic anti-cancer agents. According to previous investigations, the use of G-CSF does not have a negative impact on the survival of patients with lung cancer^32^ or ovarian cancer that are receiving chemotherapy^33^. The impact of G-CSF on the survival of cervical cancer patients has never been investigated, and thus, we cannot make any scientifically valid comments regarding the use of G-CSF for this purpose based on the results of the current study. However, as prolonged exposure to a high level of G-CSF can paradoxically enhance tumor growth, the cautious use of G-CSF is recommended.

In conclusion, we have shown that tumor G-CSF expression is an independent poor prognostic factor in cervical cancer patients that are treated with platinum-based chemotherapy. Moreover, the increased accumulation of MDSC in tumors induced by tumor-derived G-CSF is responsible for the development of cisplatin resistance in G-CSF-producing cervical cancer. We intend to prospectively evaluate our clinical findings and mechanistic hypothesis in a collaborative multi-institutional investigation involving G-CSF-producing cervical cancer patients in future.

## Methods

### Patients and clinical samples

Permission to proceed with the data acquisition and analysis was obtained from Osaka University Hospital’s institutional review board (IRB). A list of patients who were treated with platinum-based chemotherapy for recurrent or advanced cervical cancer at Osaka University Hospital from April 1996 to March 2011 was generated from our institutional tumor registry, and their clinical data were retrospectively analyzed. Cervical tumor tissue and blood samples were also collected and archived according to protocols approved by the IRB of Osaka University Hospital. All experiments were performed in accordance with guidelines and regulations approved by the IRB of Osaka University Hospital. Appropriate informed consent was obtained from each patient.

PFS was measured as the time from the diagnosis of recurrence or advanced disease to disease progression. OS was defined as the time from the diagnosis of recurrence or advanced disease to death or the last observation. The treatment-free interval was defined as the time from the end of the primary treatment to the detection of recurrence.

### Response evaluations

The response to treatment was assessed according to the Response Evaluation Criteria in Solid Tumors after every 3 cycles of each regimen. A CR was defined as the disappearance of all target and non-target lesions and the absence of new lesions on two consecutive assessments performed at least 4 weeks apart. A PR was defined as at least a 30% reduction in the sum of the longest dimensions of the target lesions on two consecutive assessments performed at least 4 weeks apart. Progressive disease was defined as a 20% increase in the sum of the longest dimensions of the target lesions or the development of new lesions. Stable disease implies that none of the above applies. The response rate was defined as the percentage of complete and partial responders.

### Reagents/antibodies

The following labeled monoclonal antibodies were used for the staining experiments: anti-human antibodies: V450-conjugated anti-CD33 (eBiosciences, San Diego, CA) and APC-conjugated anti-HLA-DR (Biolegend, San Diego, CA); anti-human/mouse antibodies: FITC-conjugated anti-CD11b (Tonbo Biosciences, San Diego, CA); anti-mouse antibodies: PE-conjugated anti-Gr-1 (R&D systems, Minneapolis, MN), APC-conjugated anti-Ly6G, and PE-conjugated anti-Ly6C (Tonbo Biosciences, San Diego, CA). Antibodies against the G-CSF receptor (G-CSFR)(Abcam, Cambridge, United Kingdom), Stat3, phospho-Stat3 (Tyr^705^) and β-actin (Cell Signaling Technology, Beverly, MA) were used for the Western blotting analysis. A neutralizing antibody against Gr-1 (RB6-8C5) was purchased from BioXCell (West Lebanon, NH). A neutralizing antibody against G-CSF and recombinant human G-CSF were kindly provided by Kyowa Hakko Kirin, Co., Ltd. (Tokyo, Japan). G-418 was purchased from Life Technology (Grand Island, NY). Cisplatin was purchased from Sigma (St. Louis, MO).

### Cell culture

ME180 cervical cancer cells were purchased from the American Type Culture Collection. The cell lines were passaged in our laboratory soon after they were received from the cell bank, before being divided and stored in liquid nitrogen vessels. Each experiment was carried out using thawed cells without further authentication. The cells were maintained in Dulbecco’s modified Eagle’s medium (DMEM) supplemented with 10% FCS.

### Clone selection

The expression vector for the mouse G-CSF gene (pCAmG-CSF) and the empty vector (pCAZ 2) used in this study, which were described previously^11^, were provided by the RIKEN BRC through the National Bio-Resource Project of the MEXT, Japan. The expression of these genes was driven by the CAG promoter, as reported previously^34^,^35^. The expression vector for the human G-CSFR (pCMV6-AC-GFP) was obtained from OriGene (Rockville, MD). Transfection was performed using Lipofectamine 2000 (Invitrogen, Carlsbad, CA) in accordance with the manufacturer’s instructions. Clonal selection was performed by adding G-418 to the medium at a final concentration of 500 μg/ml.

### Determination of G-CSF levels

The G-CSF concentrations of the serum samples were determined by enzyme-linked immunosorbent assay (ELISA) using Quantikine assay system for human G-CSF (R&D Systems, Minneapolis, MN) according to the manufacturer’s protocol.

### Reverse transcriptase polymerase chain reaction (RT-PCR)

RNA was extracted from cells using Trizol (Life Technologies, Grand Island, NY). The resultant total RNA (1 μg) was used to synthesize cDNA with ReverTraAce qPCR RT Master Mix (Toyobo, Osaka, Japan). PCR was performed using Taq PCR master mix (Qiagen, Valencia, CA) and specific primers. Amplification was performed using a Takara PCR personal-type thermal cycler (Takara, Shiga, Japan). The PCR primers were purchased from Life Technologies (Grand Island, NY). The sequences of the primers used were as follows: β-actin: forward primer, 5′-CGTGACATTAAGGAGAAGCTGTG-3′ and reverse primer, 5′-GCTCAGGAGGAGCAATGATCTTGA-3′; G-CSF: forward primer, 5′-TGAGTGTGCCACCTACAAGC-3′ and reverse primer, 5′-GACACCTCCAGGAAGCTCTG-3′; G-CSFR: forward primer, 5′-ACAAGCCGCAGCGTGGAGAAG-3′ and reverse primer, 5′-TTCTGAAGGCAGGTGGAAGGTG-3′.

### Real-time RT-PCR

Real-time RT-PCR was performed using SYBR Green PCR master mix (Applied Biosystems, Carlsbad, CA) on a Step One Plus sequence detection system (Applied Biosystems, Carlsbad, CA). Relative mRNA expression fold values were determined using standard deltaCt calculations. The PCR primers for GAPDH were purchased from Invitrogen (Carlsbad, CA), and those for Bv8 were purchased from Eurofins Operon (Huntsville, AL). The sequences of the primers used were as follows: GAPDH: forward primer, 5′-CCCTCAAGATTGTCAGCAATGC-3′ and reverse primer, 5′-GTCCTCAGTGTAGCCCAGGAT-3′; Bv8: forward primer, 5′-GCATGACAGGAGTCATCATTTT-3′ and reverse primer, 5′-AAATGGCAGGATATCAGGAAA-3′.

### Western blot analysis

The cells were lysed for 10 minutes at 4 °C. Equal amounts of protein were separated by SDS-PAGE and transferred to PVDF membranes. Western blot analyses were conducted using various specific primary antibodies. The resultant immunoblots were visualized with horseradish peroxidase (HRP)–coupled immunoglobulins using an enhanced chemiluminescence Western blotting system (PerkinElmer, Santa Clara, CA).

### Immunohistochemistry

Tumor samples were fixed in 10% neutral buffered formalin, embedded in paraffin, sectioned, and processed for immunohistochemical staining. The primary antibody used was anti-G-CSF polyclonal antibody (N-20) (Santa Cruz Biotechnology, Santa Cruz, CA). The surrounding non-neoplastic stroma served as an internal negative control for each slide, as reported previously^6^,^11^. Optical image capture was performed using PROVIS AX80 (Olympus, Tokyo, Japan). The slides were examined using a bright field microscope by two observers (K.S and E.M), who were blinded to the patients’ clinical data, and were scored semi-quantitatively. “Zero” indicated no staining, “weak” was indicative of positive staining, and “strong” represented intensely positive staining, as described in detail elsewhere^6^.

### Cell proliferation assay

The MTS assay was used to analyze the effects of cisplatin. Cervical cancer cells were plated in 96-well plates and exposed to cisplatin at different concentrations in the presence of 10% FCS. After 72 hours’ incubation, the number of surviving cells was assessed by determining the absorbance of the dissolved formazan product at 490 nm, as described by the manufacturer (Promega, Madison, WI). Cell viability is expressed as follows: absorbance of the experimental group/ absorbance of the control group.

### *In vivo* tumor studies

All of the procedures involving animals and their care were approved by the animal care and usage committee of Osaka University, in accordance with institutional and NIH guidelines. To examine the *in vivo* anti-tumor activity of cisplatin and anti-Gr-1-neutralizing antibody five- to seven-week-old BALB/c nude mice were subcutaneously inoculated with 5 × 10^6^ ME180-control or ME180-G-CSF cells in 100 μL of phosphate-buffered saline (PBS). Cisplatin was administered weekly at a dose of 4 mg/kg after the tumors reached about 50 mm^3^ in volume. Anti-Gr-1 antibody was given from the first day of cisplatin administration at a dose of 200 μg/mouse every 48 hours. Both cisplatin and anti-Gr-1 antibody were administered intraperitoneally. The splenectomy procedures were performed as reported previously^12^. Ten days after undergoing splenectomy or a sham operation, the mice were inoculated with cervical cancer cells. At the end of the experiment, the mice were killed by carbon dioxide asphyxiation, and their tumors were collected for analysis. Tumor growth was assessed in three dimensions using calipers, and tumor volume was calculated using the formula V = L × W × D × 1/2, where V is volume, L is length, W is width, and D is depth. WBC and granulocytes were counted with a VetScan HM2 automatic cell counter (Abaxis, Union City, CA).

### Isolation of MDSC

MDSC were isolated from single-cell preparations of mouse splenocytes using the MDSC isolation kit (mouse) and an MS column (Miltenyi Biotec, Auburn, CA), according to the manufacturer’s instructions. The purity of the isolated cell populations was determined by flow cytometry, and the frequency of CD11b^+^ Gr-1^+^ cells was >95%^11^.

### Detection of apoptosis

To assess the lifespan of the MDSC, cells were stained with propidium iodide and annexin V using the annexin V-FITC apoptosis detection kit (BioVision, San Francisco, CA), according to the manufacturer’s instructions. Fluorescence data were collected using flow cytometry.

### Flow cytometry

Single cell suspensions were prepared from mouse bone marrow, blood, spleen, and tumor specimens. RBCs were removed using ammonium chloride lysis buffer. To prepare human samples, peripheral blood mononuclear cells (PBMC) were separated by gradient centrifugation using Lymphoprep (Axis-Shield, Oslo, Norway). Then, cells were filtered through 40 μm nylon strainers, incubated with antibodies, and analyzed by flow cytometry. Flow cytometric data were acquired on FACSCanto II flow cytometer and analyzed using FACSDiva software (BD Biosciences, San Jose, CA). Cells that had been incubated with irrelevant isotype-matched antibodies and unstained cells served as controls.

### T cell proliferation assay

A 96-well plate was coated with 1 μg/well anti-CD3e antibody (Tonbo Biosciences, San Diego, CA). CD8 positive T cells were purified from the spleen of Balb/c mice using T cell isolation columns (R&D systems, Minneapolis, MN) according to manufacturer’s instructions. To determine the impact of MDSC on T cell proliferation, purified MDSC from the spleen of a G-CSF treated mouse were co-cultured with T cells. Cell proliferation was assayed using a cell proliferation ELISA BrdU kit (Roche Applied Science, Penzberg, Germany).

### Statistical analysis

Continuous data were compared between groups using the Student’s *t* test or Wilcoxon rank sum test. Frequency counts and proportions were compared between groups using the chi-square test or a two-tailed Fisher’s exact test, as appropriate. We compared the Kaplan–Meier curves for each subgroup with the log rank test. Cox proportional hazards regression analysis with stepwise variable selection was performed to identify significant independent predictors of OS. *P*-values of <0.05 were considered statistically significant. All analyses were performed with SAS version 9.1 for Windows (SAS Institute Inc., Cary, NC).

## Additional Information

**How to cite this article**: Kawano, M. *et al.* The significance of G-CSF expression and myeloid-derived suppressor cells in the chemoresistance of uterine cervical cancer. *Sci. Rep.* **5**, 18217; doi: 10.1038/srep18217 (2015).

## Supplementary Material

Supplementary Information

## Figures and Tables

**Table 1:** Prognostic significance of G-CSF expression.

		n	Univariate analysis			Stepwise multivariate analysis		
			HR	95%CI	P-value	HR	95%CI	P-value*
Age	<59	50	1		0.0503	1		0.0236
	60<	32	0.596	(0.346, 1.001)		0.522	(0.287, 0.918)	
Performance status	0–1	72	1		0.2130	1		0.0375
	2	10	1.613	(0.740, 3.123)		2.408	(1.056, 4.999)	
Treatment free interval	12<	59	1		0.0047	1		0.0020
(months)	<11	23	2.278	(1.275, 4.336)		2.478	(1.379, 4.743)	
Symptoms	No	51	1		0.1814			
	Yes	31	1.429	(0.843, 2.376)				
Tumor G-CSF expression	zero-weak	67	1		0.0029	1		0.0061
	strong	15	2.959	(1.484, 5.576)		2.750	(1.356, 5.297)	

^*^P-values were calculated using the two-sided Wald test in the Cox proportional hazard model.

G-CSF = Granulocyte-colony stimulating factor; HR = hazard ratio; CI = confidence interval.
