# Supplementary material for: The significance of G-CSF expression and myeloid-derived suppressor cells in the chemoresistance of uterine cervical cancer
Source: Sci Rep. 2015 Dec 15;5:18217. doi: 10.1038/srep18217 (PMC4678366; doi:10.1038/srep18217)
Supplement: Supplementary Information [file srep18217-s1.pdf]

The significance of G-CSF expression and myeloid-derived suppressor cells in the chemoresistance of uterine cervical cancer

Mahiru Kawano, Seiji Mabuchi, Yuri Matsumoto, Tomoyuki Sasano, Ryoko Takahashi, Hiromasa Kuroda, Katsumi Kozasa, Kae Hashimoto, Aki Isobe, Kenjiro Sawada, Toshimitsu Hamasaki, Eiichi Morii, Tadashi Kimura

Supplementary Table S1. Patients' characteristics

			All patients<br>Number of patients (%)<br>(n=82)	G-CSF strong<br>Number of patients (%)<br>(n=15)	G-CSF zero-weak<br>Number of patients (%)<br>(n=67)	P-value*
Age	Mean (SD)		55.4 (12.5)	55.3 (10.7)	55.5 (12.9)	0.8524
	≤59		50 (61.0)	11 (73.3)	39 (58.2)	0.2679
	60≤		32 (39.0)	4 (26.7)	28 (41.8)	
Disease status and FIGO stage	Recurrent	IA-IIA	24 (29.3)	7 (46.7)	17 (25.4)	0.1105
		IIB-IVA	42 (51.2)	4 (26.7)	38 (56.7)	
	Advanced	IVB	16 (19.5)	4 (26.7)	12 (17.9)	
Site of disease	Pelvic		21 (25.6)	6 (40.0)	15 (22.4)	0.2686
	Distant		19 (23.2)	4 (26.7)	15 (22.4)	
	Pelvic + Distant		42 (51.2)	5 (33.3)	37 (55.2)	
Histology	SCC		51 (62.2)	9 (60.0)	42 (62.7)	0.0621
	A or AS		26 (31.7)	3 (20.0)	23 (34.3)	
	Others		5 (6.1)	3 (20.0)	2 (2.9)	
Performance status	0-1		72 (87.8)	12 (80.0)	60 (89.6)	0.3339
	2		10 (12.2)	3 (20.0)	7 (10.5)	
Symptoms	Yes		31 (37.8)	5 (33.3)	26 (38.8)	0.6908
	No		51 (62.2)	10 (66.7)	41 (61.2)	
Treatment free interval (month)	Median (Min-max)		7 (0-132)	5 (0-132)	7 (0-91)	0.1411†
	≤12		59 (72.0)	11 (73.3)	48 (71.6)	0.8947
	11≤		23 (28.0)	4 (26.7)	19 (28.4)	
Prior systemic chemotherapy	Yes		10 (12.2)	2 (13.3)	8 (11.9)	0.8827
	No		72 (87.8)	13 (86.7)	59 (88.1)	
Chemotherapy regimen	CBDCA+PTX		60 (73.2)	8 (53.3)	52 (77.6)	0.0577
	CBDCA+PTX+EPI		13 (15.9)	3 (20.0)	10 (14.9)	
	CDGP+5-FU		5 (6.1)	3 (20.0)	2 (3.0)	
	Others		4 (4.9)	1 (6.7)	3 (4.5)	

* Wilcoxon Rank Sum tests were used for continuous variables (Age). Chi-square test and Fisher's Exact test were used for categorical variables (Age, Disease status, Site of disease, Histology, Performance status, Symptoms, Treatment free interval, Prior systemic chemotherapy and Chemotherapy regimen). All statistical tests were two-sided.

†Median test

G-CSF = granulocyte-colony stimulating factor; SCC = squamous cell carcinoma; A = adenocarcinoma; AS = adenosquamous carcinoma; CBDCA = carboplatin; PTX = paclitaxel; EPI = epirubicin; CDGP = nedaplatin; 5-FU = fluorouracil.

Supplementary Figure S1

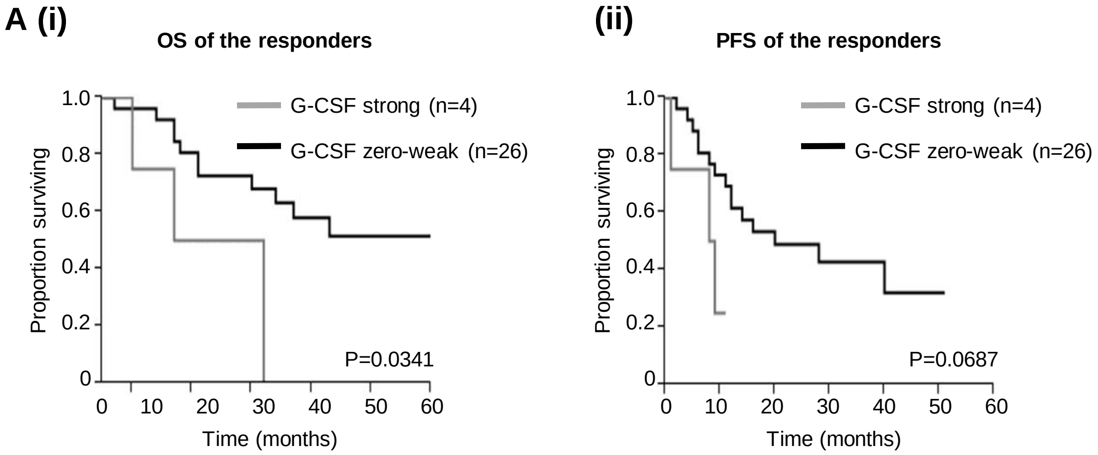

Supplementary Figure S1. Survival analysis of the responders

A, Kaplan–Meier estimates survival of the responders according to G-CSF immunoreactivity. Log-rank test was used to determine statistical significance. (i) Overall survival (OS). (ii) Progression free survival (PFS).

Supplementary Figure S2

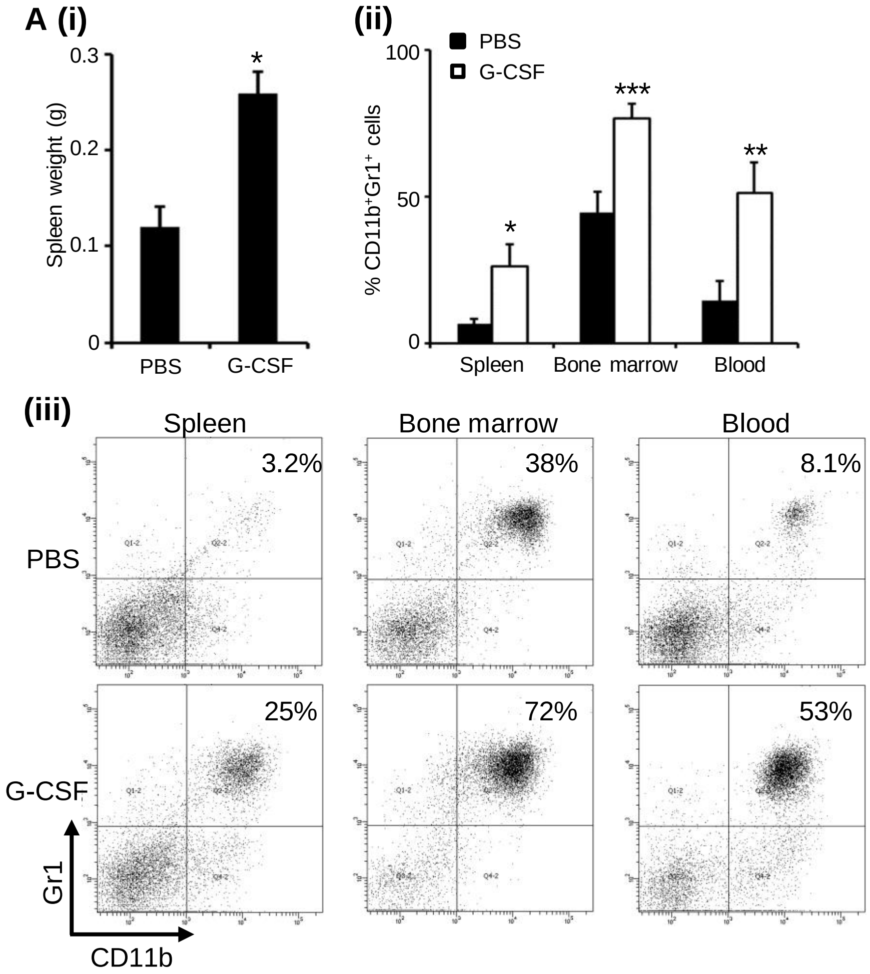

Supplementary Figure S2. G-CSF-induced MDSC

A, Effects of exogenous G-CSF treatment on the spleen weight and the frequency of MDSC. Mice were subcutaneously treated with 10 μg recombinant human G-CSF (n=5) or PBS (n=5) for three consecutive days. The spleen, bone marrow, and blood were harvested for evaluation. (i) Spleen weight. Bars, SD. *, P<0.05, Wilcoxon Rank sum test. (ii) MDSC populations were assessed by flow cytometry. Bars, SD. *, P<0.05, **, P<0.01, ***, P<0.001, Two-sided Student *t* test. (iii) Representative dot plot.

Supplementary Figure S3

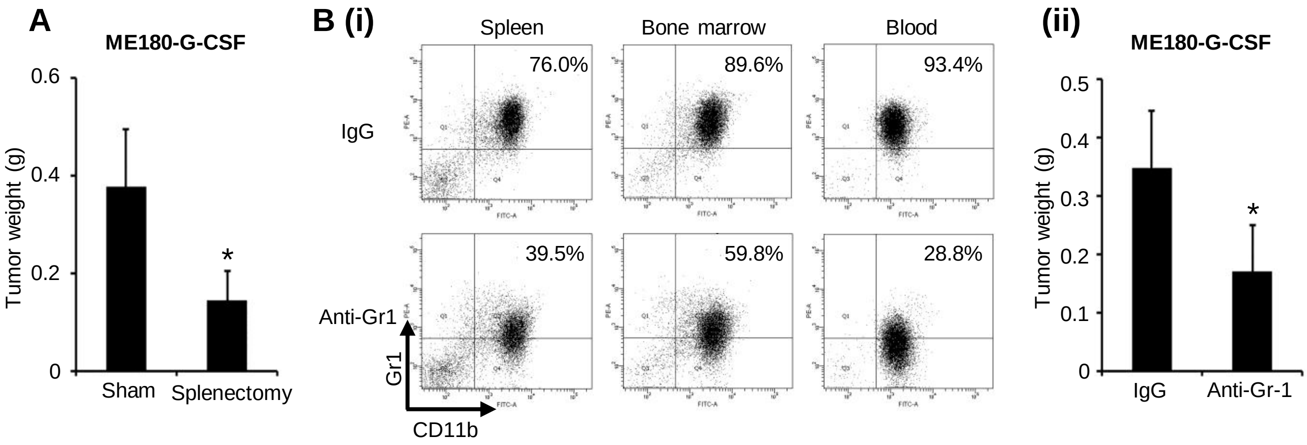

Supplementary Figure S3. MDSC depletion and cisplatin-sensitivity

A, Effects of spleen removal on the tumor weight of G-CSF-producing cervical cancer. Mice that had undergone splenectomy or sham surgery were inoculated with ME180-G-CSF (n=5 for each group) and treated with cisplatin. Bars, SD. *, P<0.05, Wilcoxon Rank sum test. **B,** Effects of anti-Gr-1-neutralizing antibody on the MDSC frequency and the tumor weight of G-CSF-producing cervical cancer. Mice carrying ME180-G-CSF-derived tumors were treated with cisplatin in combination with anti-Gr-1-neutralizing antibody or IgG (n=5 for each group). (i) MDSC population. Their spleens, bone marrow, and blood were collected and assayed for MDSC by flow cytometry. Representative dot plot. (ii) Tumor weight. Bars, SD. *, P<0.05, Wilcoxon Rank sum test.
